# Fatty acid status and behavioural symptoms of Attention Deficit Hyperactivity Disorder in adolescents: A case-control study

**DOI:** 10.1186/1475-2891-7-8

**Published:** 2008-02-14

**Authors:** Ashley L Colter, Caroline Cutler, Kelly Anne Meckling

**Affiliations:** 1 Department of Human Health and Nutritional Sciences, University of Guelph, Guelph, ON, N1G 2W1, Canada

## Abstract

**Background:**

Most studies of Attention-deficit hyperactivity disorder (ADHD) have focused on either young children or older adults. The current study compared 11 ADHD adolescents with 12 age-matched controls. The purpose was to examine differences in dietary intake, particularly of essential fatty acids, and determine whether this could explain the typical abnormalities in red blood cell fatty acids observed in previous studies of young children. A secondary purpose was to determine if there were relationships between circulating concentrations of essential fatty acids and specific ADHD behaviours as measured by the Conners' Parent Rating Scale (CPRS-L).

**Methods:**

Eleven ADHD adolescents and twelve age-matched controls were recruited through newspaper ads, posters and a university website. ADHD diagnosis was confirmed by medical practitioners according to DSM-IV criteria. Blood, dietary intake information as well as behavioural assessments were completed.

**Results:**

Results showed that ADHD adolescents consumed more energy and fat than controls but had similar anthropometry. ADHD children consumed equivalent amounts of omega-3 and omega-6 fatty acids to controls, however they had significantly lower levels of docosahexaenoic acid (DHA, 22:6n-3) and total omega-3 fatty acids, higher omega-6 fatty acids and a lower ratio of n-3:n-6 fatty acids than control subjects. In addition, low omega-3 status correlated with higher scores on several Conners' behavioural scales.

**Conclusion:**

These data suggest that adolescents with ADHD continue to display abnormal essential fatty acid profiles that are often observed in younger children and distinctly different from normal controls of similar age. Further these red blood cell fatty acid differences are not explained by differences in intake. This suggests that there are metabolic differences in fatty acid handling between ADHD adolescents and normal controls. The value of omega-3 supplements to improve fatty acid profiles and possibly behaviours associated with ADHD, need to be examined.

## Background

Attention deficit hyperactivity disorder (ADHD) is primarily characterized by a "persistent pattern of inattention and/or hyperactivity-impulsivity that is more frequent and severe than is typically observed in individuals at a comparable level of development" [1,2]. The American Psychiatric Association estimates that 3–5% of school aged children have ADHD (DSM-IV), while other sources report higher prevalence rates ranging from 5–13% [3-6]. ADHD is the most common psychiatric disorder in children and is diagnosed in males two to nine times as often as in females. ADHD shows high comorbidity with several other conditions including learning differences, oppositional defiance disorder (ODD), obsessive compulsive disorder (OCD) and depression [7,8] For up to 60% of these children, ADHD symptoms and difficulties will persist into adulthood [9,10].

The cause of ADHD is generally acknowledged to be multifactorial, involving both biological and environmental influence [2,11]. In the past two decades, there has been an increasing focus particularly on the effects of diet in hyperactivity in children. Researchers have reported that various aspects of a child's diet including food additives, refined sugars, food allergies, minerals and fatty acid metabolism may have adverse effects on behaviour[7,12,13]. While there is no definitive proof that any of these is responsible for the spectrum of ADHD symptoms, there is a compelling argument for a role for long-chain polyunsaturated fatty acids.

The processes of elongation and desaturation occur mainly in the liver, but also in the central nervous system, placenta, glial tissue and choroid plexus vasculature[14]. Within the brain, four fatty acids are particularly important; dihomogammalinolenic acid (20:3n-6, DGLA), arachidonic acid (20:4n-6, AA), eicosapentaenoic acid (20:5n-3, EPA) and docosahexaenoic acid (22:6n-3). AA and DHA play a major structural role in neuronal membranes and make up 20% of the dry mass of the brain[11]. In addition the eicosanoid and other fatty acid metabolites of various LC-PUFAs, though at much lower concentrations, could play important roles in brain function [15-19]. EPA and DGLA play a more minor structural role but are also crucial for normal brain function. Since optimal requirements are not fully known, definitive dietary reference intakes (DRIs) for the omega-3 and omega-6 fatty acids have not yet been determined[20]. However, Petrie and colleagues published recommendations for adequate intake (AI) for boys 9–16 yr as 12–16 g linoleic acid (LA)/d and 1.2–1.6 g α-linolenic acid (ALA)/d. For girls the corresponding amounts were 10–11 LA g/d and 1.0–1.1 ALA g/d[21]. In order to ensure the best biological functions, Bjerve suggests an intake of 900 mg/d EPA and 400 mg/d DHA[22].

A number of the physical and behavioural symptoms of essential fatty acid deficiency mimic some of the symptoms described in typical ADHA patients, therefore it is conceivable, that either dietary deficiency of omega-3 fatty acids, or altered metabolic handling of these fatty acids, could contribute to the abnormalities observed in those affected by ADHD. Several studies have examined fatty acid status in patients with ADHD, but only recently have researchers begun to examine efficacy of high dose supplementation on ADHD behaviours [13,23-25].

The purpose of the present study was to compare several parameters in an adolescent ADHD population versus an adolescent control population. Parameters included comparisons of dietary patterns between the groups based on 7-day diet records and analysis of red blood cell fatty acid composition and serum hormone levels as well as the frequency of symptoms of fatty acid deficiency and ADHD-associated behaviours in both populations.

## Experimental methods

### Subject selection

This study was approved by the Research Ethics Board at the University of Guelph.

Adolescent males and females aged ten to sixteen years were recruited from the City of Guelph and surrounding area starting in March 2004 via flyers and local advertisements. Approximately 45 parents and/or subjects contacted the study coordinator for further information regarding the study protocol. Of these, 23 subjects and their families agreed to participate and gave informed consent. The main reason for declining to participate was a result of the child or adolescent's refusal to have blood taken.

In all there were 11 subjects with a confirmed physician diagnosis of ADHD according to DSM-IV criterion and 12 control subjects without a diagnosis of ADHD that acted as participants. One subject in the control group completed study visit #1 before dropping out of the study due to lack of interest and three subjects in the control group participated in a single visit, condensed study protocol, due to time constraints. The remaining 19 subjects participated in the entire protocol.

### Experimental protocol

All study visits occurred at the Human Nutraceutical Research Unit. All subject visits were scheduled in advance via email or telephone and occurred on weekday mornings between the hours of 7 and 11 am. Study visits for male and pre-menstrual female subjects were coordinated on the basis of convenience for the participants. Study visits for female subjects who had begun menstruation were coordinated to occur on or as close menstrual cycle day 5. It was mandatory that a parent or guardian be on campus with the subject for visit one. All subjects were required to fast for a period of at least 8 h prior to study visits and were allowed to consume water only during this period.

### Study visit #1

Following introductions, the subject and guardian were invited to sit with the study coordinator to go through experimental details and protocols and sign a consent form. Following this, a Subject Health Questionnaire and Conners' Parent Rating Scale (CPRS:L) were explained and presented to the guardian for completion. During this visit, a variety of baseline measurements were taken for each subject. Height (without shoes) was measured in centimeters (cm) and weight (kg) in light clothing recorded (Acculab SV-100, Haverhill, MA). Following a 5 min rest period, blood pressure and pulse were measured using a battery operated portable machine (Lifesource, Milpitas, CA) and were recorded in systole over diastole and beats per minute (bpm), respectively. Next, body composition was estimated using bioelectrical impedance analysis (BIA, BodyStat 1500, British Isles). Following body composition measures, a fasting venous blood sample was taken for further analysis (four untreated, four in heparinized tubes, Vacutainer, Becton Dickinson, NJ). Following blood collection, subjects were provided with unlimited beverages and a small breakfast.

The purpose and protocol for the completion of the 7-day dietary record was then explained to both the subjects and their guardians. Subjects were instructed to keep their usual dietary habits and the importance of accurate completion was stressed. Subjects were given a study package to take home with them, which included 5 two-sided dietary record sheets, written explanation and details of how to complete them, and reference sheets for common food sizes. Before leaving a tentative return date was scheduled for 4–6 weeks later.

### Behaviour assessment

The revised Conners' Parent Rating Scale long version (CPRS-R:L) was administered during visit #1 of the study for all subjects and completed by the attending parent or guardian. Assessment of 80 common problems was based on the child's behaviour in the preceding month and they were asked to circle the best answer for each item. All parent-rating scores were converted to T-scores through the use of the CPRS:L sheet for score profiling. The scale was used to assess not only ADHD, but also problems with conduct, cognition, family relationships, emotional issues, anger management, and/or anxiety.

### Study visit #2

Four to six weeks following the first visit, a second study visit was coordinated. Subjects were required to fast for a period of at least 8 hours as with visit one, and the four tubes of blood were taken as close to arrival time as possible. Subjects were again provided with a snack following blood collection. Following this, the dietary record sheets were collected and the distribution and access to the study results were explained to all subjects and their guardian. Prior to departure, the subject was presented with a $25 gift certificate for participation. Subjects who participated in the condensed study protocol, which included one visit only, were presented with a $15 gift certificate. All study visits occurred between June 2004 and August 2005.

### Diet analysis

All subjects recorded their daily diet consumption for seven consecutive days between study visit 1 and 2. On study visit 2 these pages were submitted to the investigator. Diet analysis was performed using the computer-based program Food Processor^© ^(Version 7.11, ESHA Research, Oregon). All food and beverage items ingested over the seven day period were entered into the program, and nutrition values were averaged to a per day basis.

### Blood analysis

Following collection, the red top tubes were placed at room temperature for at least 15 min to allow clotting. Heparinized tubes were immediately placed on ice. All tubes were centrifuged for 20 min at 500 × g. The serum and plasma fractions were collected by pipette and deposited into labeled aliquot tubes for storage. Red blood cell fractions were prepared following removal of plasma and white blood cells from sodium heparinized samples and tubes were filled to the top with 0.9% sodium chloride saline solution (Abbott Laboratories Ltd., Quebec). The contents were mixed well by inversion and the tube was then centrifuged for 20 min at 500 × g. Following this wash, the upper saline layer was discarded and the red blood cell fraction was collected and pipetted into labeled aliquot tubes. All aliquot tubes were stored at -20 degrees for a maximum of 5 days and were then transferred to a -80 freezer until analysis.

### Red blood cell fatty acid analysis

Following chloroform:methanol extraction, phospholipids were separated from other lipids my thin layer chromatography and methylated fatty acids determined by an outside commercial laboratory (Lipid Analytical Laboratories, Guelph, ON). Fatty acids were measured in RBC blood samples from both study visits 1 and 2, when available, and values were averaged for the purpose of statistical analyses. Fatty acid values are presented as percentage of molecular weight.

### Statistical Analysis

Statistical significance was accepted at p < 0.05, using a two-tailed test. All statistical analysis was performed using SPSS 10.0 for Windows Student Version. Statistical analyses performed included independent samples t-test for means comparisons and bi-variate Pearson's correlations. Data is reported as mean ± standard deviation (SD), and in some cases, followed by the range of values in parentheses. All data were normally distributed and the differences in SDs between groups were not significantly different.

## Results

Subject characteristics at visit #1 are given in Table 1. There were no significant differences between the groups for age and sex characteristics or anthropometric measures. A health questionnaire was filled out at the start of the study by the parent/guardian for each subject. The questionnaire collected information regarding ADHD diagnosis, medication use, co-morbid disorders/conditions, vitamin/supplement use, family history of ADHD, allergies, duration of breastfeeding and prevalence of fatty acid deficiency symptoms. Six of eleven subjects in the ADHD group were taking medications (55%), five of eleven presented with a co-morbid learning disorder (45%), and eight of eleven reported a history of ADHD within the family (73%). These three variables were significantly different from the control group with p = 0.016, p = 0.016 and p = 0.001, respectively.

**Table 1:** Baseline subject characteristics and anthopometric measurements

	**ADHD Group (n = 11)**	**Control Group (n = 12)**
**Variable**	**Mean ± SD**	**Mean ± SD**
Sex, n male/female	9/2	6/6
Age, years ± SD	13.6 ± 2.2	14.2 ± 1.9
Age range, years	10.4–16.4	11.3–16.6
Height (cm)	163.5 ± 12.2	160.5 ± 8.4
Weight (kg)	52.7 ± 15.5	49.5 ± 12.2
BMI (kg/m^2^)	19.3 ± 3.8	19.0 ± 3.2
Lean weight (kg)	43.6 ± 16.4	38.3 ± 8.4
% lean mass	80.6	77.7
Fat weight (kg)	9.1 ± 3.6	11.2 ± 4.8
% fat mass	19.4	22.3
Systolic blood pressure (mmHg)	107.0 ± 17.7	109.6 ± 9.7
Diastolic blood pressure (mmHg)	66.6 ± 8.2	66.5 ± 7.1
Heart Rate (bpm)	66.5 ± 8.7	72.8 ± 14.4

The duration of breastfeeding in infancy was also reported. Subjects in the ADHD group were breastfed an average of 4.14 ± 2.6 (0–7) months, whereas subjects in the control group were breastfeed an average of 10.4 ± 10.9 (3–39) months. With respect to symptoms of fatty acid deficiency, subjects in the ADHD group reported an average of 2.4 ± 3.5 (0–9) symptoms versus 1.6 ± 1.6 (0–4) symptoms in the control group. Neither of these differences between groups was significant (p = 0.077 and p = 0.461, respectively).

The ADHD group was also more likely to have allergies (27% vs. 8%) and less likely to be taking vitamins (18% vs. 50%) when compared to the control group. Again, neither of these differences were significant.

### Behaviour assessment

**Figure 1:**
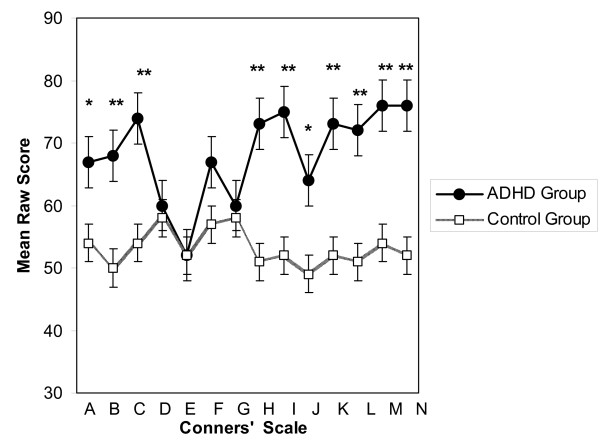
**Conners' Parent Rating Scale (CPRS:L) mean T scores (with mean standard error) for ADHD group versus controls.** CPRS:L scales; A: Oppositional, B: Cognitive Problems/Inattention, C: Hyperactivity, D: Anxious-Shy, E: Perfectionism, F: Social Problems, G: Psychosomatic, H: Conners' ADHD Index, I: CGI Restlessness-Impulsive, J: CGI Emotional Lability, K: CGI Total, L: DSM-IV Inattentive, M: DSM-IV Hyperactive-Impulsive, N: DSM-IV Total. ADHD group presented with significantly higher mean raw scores on Scales A-C and H-N, for a total of 10/14 scales. Significantly different from control group ** p < 0.01, * p < 0.04.

Analysis of the Conners' Parent Rating Scales Long Version (CPRS:L) revealed several significant differences between the ADHD and control groups (Table 2). When compared to the control group, the ADHD group presented with significantly higher mean T scores on ten of the fourteen scales included in the assessment (p < 0.05) (Figure 1). These included measures for oppositional behaviours, cognitive problems and inattention, restlessness and impulsivity, hyperactivity, emotional lability and overall problematic behaviour. Children were also identified as 'at risk' through scores on the ADHD index, while also being assessed on scales directly related to DSM-IV criteria including inattentive, hyperactive-impulsive and total DSM scores.

**Table 2:** Behaviour Assessment Data

**Conners' Rating**	**ADHD Group (n = 11)**	**Control Group (n = 12)**
**Scale: Measurement**	**Mean T Score ± SD (range)**	**Mean T Score ± SD (range)**
A: Oppositional	66.6 ± 15.8 (44–90) *	53.8 ± 8.3 (39–66)
B: Cognitive Problems/Inattention	68.0 ± 9.7 (56–84) **	50.1 ± 7.5 (43–66)
C: Hyperactivity	73.8 ± 12.8 (49–90) **	53.8 ± 10.6 (44–77)
D: Anxious-Shy	59.8 ± 17.6 (42–90)	58.3 ± 14.4 (42–90)
E: Perfectionism	51.9 ± 14.5 (40–82)	51.6 ± 9.9 (41–70)
F: Social Problems	67.4 ± 15.7 (45–90)	56.9 ± 16.8 (45–90)
G: Psychosomatic	59.6 ± 14.7 (42–90)	57.9 ± 13.4 (42–83)
H: Conners' ADHD Index	73.2 ± 10.6 (55–90) **	51.2 ± 10.8 (42–79)
I: CGI Restless-Impulsive	74.7 ± 11.7 (54–89) **	52.4 ± 8.6 (43–70)
J: CGI Emotional Lability	64.1 ± 19.5 (42–90) *	49.3 ± 7.9 (41–65)
K: CGI Total	72.6 ± 13.5 (51–90) **	51.8 ± 9.1 (42–69)
L: DSM-IV Inattentive	71.6 ± 10.0 (58–90) **	50.7 ± 7.8 (43–69)
M: DSM-IV Hyperactive-Impulsive	76.0 ± 14.1 (48–90) **	53.7 ± 10.8 (43–71)
N: DSM-IV Total	76.2 ± 11.3 (55–90) **	52.1 ± 9.0 (42–70)

Significantly different from control subjects ** p < 0.01, * p < 0.04

### Diet analysis

Analysis of seven-day dietary records from subjects (ADHD n = 11, Control n = 8) revealed several differences between intake patterns (Table 3, not all data is shown). The ADHD group consumed significantly more calories (2652 ± 458 vs. 2051 ± 407, p = 0.009), more protein (91 ± 19 g vs. 73 ± 17 g, p = 0.049) and more carbohydrates (357 ± 68 g vs. 271 ± 60 g, p = 0.011) per day when compared to the control group. The ADHD group also consumed significantly more total fat (99 ± 22 g vs. 77 ± 14 g, p = 0.013), saturated fats (38 ± 9 g vs. 28 ± 8 g, p = 0.027) and trans fatty acids (3.6 ± 3.1 g vs. 1.3 ± 0.7 g, p = 0.038) per day. There was also a trend toward higher monounsaturated fat consumption in the ADHD group (31 ± 9 g vs. 24 ± 6 g), however this difference was not significance (p = 0.077). There were no significant differences in mean consumption of total n-3 fatty acids, ALA, EPA or DHA between the two groups. Furthermore, there were no differences in mean consumption of total n-6 fatty acids, LA or AA. Calculated n-6: n-3 ratios revealed values of 9.47 in the ADHD group and 9.03 in the control group. These ratios were not significantly different from one another.

**Table 3:** Diet Record Analysis Data

**Dietary Variable**	**ADHD Group (n = 11)**	**Control Group (n = 8)**	**p value**
**Total Energy (kcal)**	2651.0 ± 458.1 *	2051.4 ± 407.0	0.009
**Protein (g)**	90.9 ± 19.0 *	73.0 ± 17.0	0.049
**Carbohydrates (g)**	356.9 ± 68.1 *	271.1 ± 59.6	0.011
**Total Fat (g)**	99.4 ± 21.8 *	76.7 ± 13.6	0.013
Saturated Fat (g)	38.1 ± 9.1 *	28.3 ± 8.2	0.027
Monounsaturated Fat (g)	30.9 ± 8.6	24.1 ± 6.4	NS
Polyunsaturated Fat (g)	10.8 ± 4.0	9.7 ± 4.3	NS
**Total omega-3 (g)**	0.72 ± 0.31	0.76 ± 0.37	NS
Alpha Linolenic Acid (g)	0.66 ± 0.28	0.71 ± 0.36	NS
Eicosapentaenoic Acid (mg)	22 ± 54	14 ± 17	NS
Docosahexaenoic Acid (mg)	31 ± 53	39 ± 47	NS
**Total omega-6 (g)**	6.82 ± 3.79	6.86 ± 3.85	NS
Linoleic Acid (g)	6.78 ± 3.79	6.81 ± 3.84	NS
Arachidonic Acid (mg)	38 ± 19	51 ± 35	NS
**omega-6: omega 3**	9.47	9.03	NS
**Vitamin B1 (mg)**	1.8 ± 0.3 *	1.4 ± 0.4	0.019
**Vitamin B2 (mg)**	2.3 ± 0.7 *	1.5 ± 0.4	0.01
**Iron (mg)**	21.7 ± 7.0 *	12.5 ± 3.4	0.002
**Sodium (mg)**	4001 ± 931*	3119 ± 755	0.042
**Calcium (mg)**	1050 ± 282	798 ± 239	NS
**Zinc (mg)**	13 ± 5	9 ± 3	NS

Data represents daily consumption as averaged from 7-day diet records. Values are reported as mean ± SD. * Significantly different from control group (p < 0.05)

The ADHD group consumed significantly greater amounts of vitamin B1 (1.84 ± 0.34 mg vs. 1.42 ± 0.37 mg), vitamin B2 (2.34 ± 0.70 mg vs. 1.54 ± 0.36 mg), iron (21.66 ± 7.04 mg vs. 12.52 ± 3.43 mg) and sodium (4001 ± 931 mg vs. 3119 ± 756 mg) in their diets when compared to controls (p < 0.05). There were also trends toward increased calcium and zinc intake in the ADHD group when compared to the control group however, these differences were not significant.

**Figure 2:**
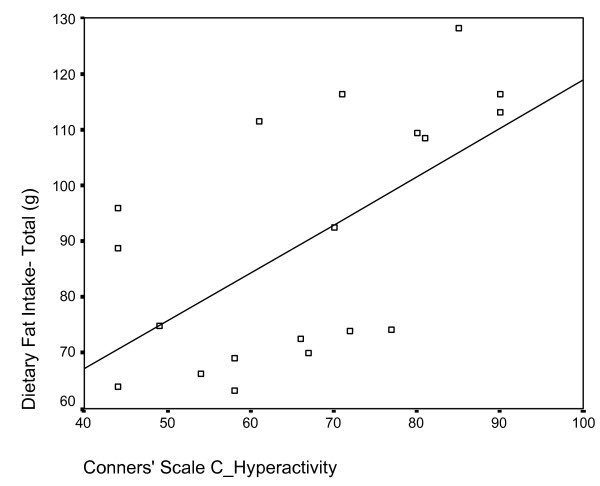
Scatterplot of dietary fat intake (y-axis) and mean raw score on Conners' hyperactivity scale (x-axis). Significant positive correlation r = .606, p = 0.003.

**Figure 3:**
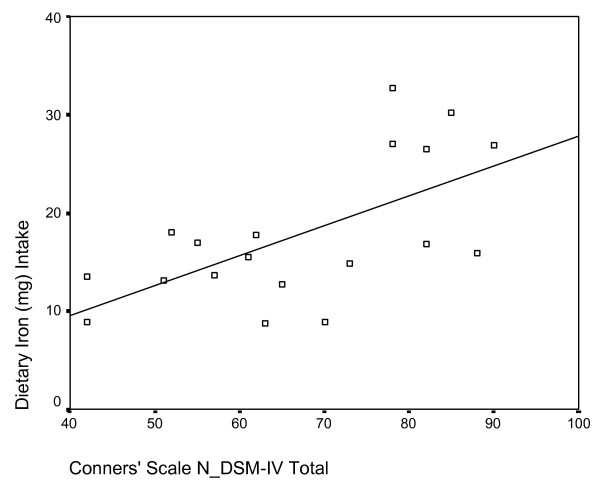
Scatterplot of dietary iron intake (y-axis) and mean raw score on Conners' DSM-IV total scale (x-axis). Significant positive correlation r = .496, p = 0.015.

Pearson correlations between diet variables and CPRS:L scale rating revealed several positive and significant relationships. Total energy intake was positively correlated with scores for oppositional and hyperactive behaviours (p < 0.01), in addition to restlessness, problematic behaviour and DSM-IV total (p < 0.05). Saturated fat and total fat intakes displayed a significant positive correlation to scales measuring oppositional, hyperactive (Figure 2) and problematic behaviours (p < 0.01), as well as DSM-IV total score (p < 0.05). Iron intake was also positively correlated with the cognitive problem, DSM-IV inattentive, DSM-IV total (Figure 3), problematic behaviour and restlessness scales (p < 0.01), in addition to the oppositional, hyperactivity and ADHD index scales (p < 0.05). Finally, intake of sodium was positively correlated with the hyperactivity and restlessness scales (p < 0.01), and with the oppositional, problematic behaviour, ADHD index, DSM inattentive and DSM total scales (p < 0.05).

### Fatty acid analysis

Phospholipid analysis of red blood cell samples was performed and all fatty acids were reported as a mol percentage of total membrane fatty acids (Table 4, not all data is shown). When compared to controls, the ADHD group presented with significantly lower DHA (3.12 ± 0.75 vs. 4.39 ± 1.34, p = 0.012) and total n-3 fatty acids (5.79 ± 1.39 vs. 7.42 ± 1.64, p = 0.018). The ratio of n-3 fatty acids to n-6 fatty acids was also significantly lower in the ADHD group (0.17 ± 0.04 vs. 0.23 ± 0.06, p = 0.017). The ADHD group also presented with elevated LA (13.26 ± 0.95 vs. 12.02 ± 2.14), total n-6 (33.33 ± 1.83 vs. 32.51 ± 1.59) and total saturated fatty acid levels when compared to the control group however; none of these latter differences were significant.

**Table 4:** Red Blood Cell Fatty Acid Analysis Data

	**ADHD (n = 11)**	**Control (n = 12)**
**Total omega-3 Fatty Acids**	5.79 ± 1.39 †	7.42 ± 1.64
Alpha-Linolenic Acid (18:3 n-3)	0.16 ± 0.07	0.13 ± 0.09
Stearidonic Acid (18:4 n-3)	0.07 ± 0.04	0.07 ± 0.04
20:3 n-3	0.02 ± 0.01	0.02 ± 0.01
Eicosatetraenoic Acid (20:4 n-3)	0.05 ± 0.05	0.05 ± 0.04
Eicosapentaenoic Acid (20:5 n-3)	0.51 ± 0.21	0.64 ± 0.24
Docosapentaenoic Acid (22:5 n-3)	1.88 ± 0.71	2.03 ± 0.56
Docosahexaenoic Acid (22:6 n-3)	3.12 ± 0.75 †	4.39 ± 1.34
		
**Total omega-6 Fatty Acids**	33.33 ± 1.83	32.51 ± 1.59
Linoleic Acid (18:2 n-6)	13.26 ± 0.95 *	12.02 ± 2.14
Gamma-Linolenic Acid (18:3 n-6)	0.05 ± 0.03	0.06 ± 0.03
Eicosadienoic Acid (20:2 n-6)	0.15 ± 0.07	0.14 ± 0.09
Dihomo-gamma-linolenic Acid (20:3 n-6)	1.69 ± 0.20 *	1.84 ± 0.51
Arachidonic Acid (20:4 n-6)	14.51 ± 1.67	14.73 ± 1.48
Docosadienoic Acid (22:2 n-6)	0.05 ± 0.03	0.04 ± 0.06
Adrenic Acid (22:4 n-6)	3.61 ± 1.33	3.63 ± 0.72
Docosapentanoic Acid (22:5 n-6)	0.03 ± 0.03	0.07 ± 0.01
		
n-3: n-6 Ratio	0.17 ± 0.04 †	0.23 ± 0.06
n-6: n-3 Ratio	5.86 ± 2.04 *	4.64 ± 1.27
AA/EPA	31.61 ± 8.91	26.04 ± 10.27
		
Total Saturated Fatty Acids	39.38 ± 1.79	39.26 ± 1.16
Total Monounsaturated Fatty Acids	21.51 ± 1.15	20.81 ± 1.15
Total Polyunsaturated Fatty Acids	39.11 ± 2.48	39.93 ± 1.76

Values are means +/- standard deviation and given as a mol % of total fatty acids. † Significant difference between groups (p < 0.02), * Difference between groups (p = 0.095)

**Figure 4:**
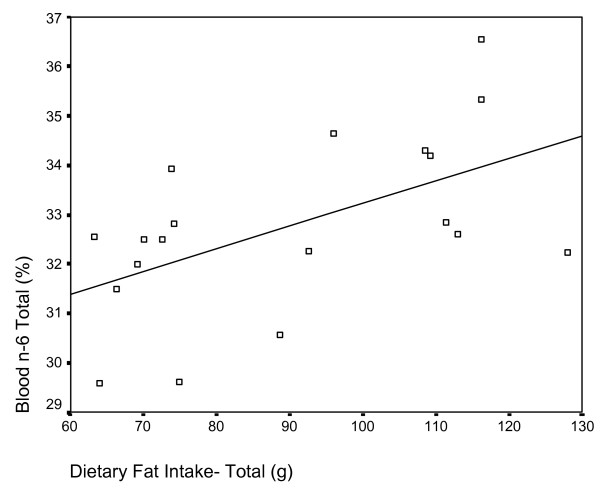
Scatterplot of red blood cell total n-6 content (y-axis) and dietary fat intake (x-axis). Significant positive correlation r = .552, p = 0.007.

**Figure 5:**
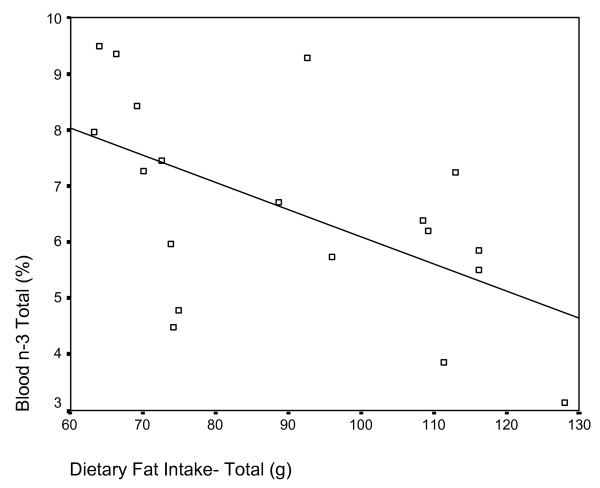
Scatterplot of red blood cell total n-3 content (y-axis) and dietary fat intake. Significant negative correlation r = -.570, p = 0.005.

Analysis of the relationship between red blood cell content and diet variables identified multiple significant correlations. Total caloric intake was positively correlated with total n-6 red blood cell content (r = .451, p = 0.026) and negatively correlated with DHA (r = -.491, p = 0.016), total n-3 (r = -.509, p = 0.013) and the n-3: n-6 ratio (r = -.544, p = 0.008). Similarly, total dietary fat intake was positively correlated with total n-6 red blood cell content (r = .552, p = 0.007) (Figure 4) and negatively correlated with DHA (r = -.532, p = 0.010), total n-3 (r = -.570, p = 0.005) (Figure 5) and the n-3: n-6 ratio (r = -.609, p = 0.003). Saturated fat and sodium intakes displayed similar trends with respect to correlation to blood fatty acid parameters.

**Figure 6:**
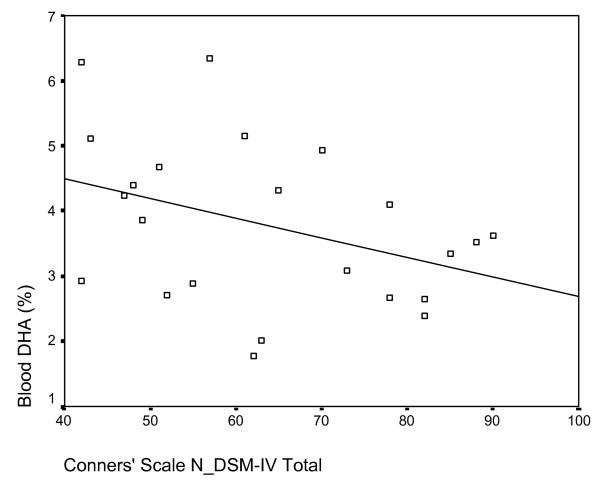
Scatterplot of red blood cell DHA content (y-axis) and Conners' DSM-IV total score (x-axis). Significant negative correlation r = -.378, p = 0.037.

There were several significant correlations between specific red blood cell parameters and Conners' scale ratings. Red blood cell DHA content was negatively correlated with scale ratings for oppositional behaviour, hyperactivity, cognitive problems, restlessness, problematic behaviour, DSM-IV inattention and DSM-IV total (p < 0.05) (Figure 6). Total n-3 content was negatively correlated with the Conners' scale for restlessness (p < 0.05) and the n-3: n-6 ratio was negatively related to the oppositional, restlessness and problematic behaviour scales (p < 0.05). Finally, total red blood cell n-6 content was positively correlated with the oppositional, restlessness, problematic behaviour, DSM-IV inattentive, DSM-IV total and ADHD index scales (p < 0.05).

## Discussion

Most clinical studies have focused on fatty acid and other abnormalities associated with ADHD in children (6–12 years) and adult populations (18–65 years) [25-28]. One recent study used a slightly older population where the average age of subjects was 11 years [24]and in the current study our subjects had a mean age of 14 years. The primary objective of this study was to determine whether abnormalities typically observed in younger and older populations are also observed in adolescents with ADHD when compared to controls.

The Conners' Parent Rating Scale (CPRS:L) was utilized for behaviour assessment in this study. The CPRS:L is well respected and has been used widely among studies assessing ADHD behaviours in children [28-32]. The Conners' scale used provides an appropriate instrument for measuring behaviours in youths aged 3 to 17 and conveys information that corresponds to the official ADHD criteria in the DSM-IV [33]. In general, T-scores of 65 and above are usually taken to indicate a clinically significant problem. In this study, the ADHD group presented with significantly higher mean T-scores than the control group on ten of the fourteen scales on the CPRS:L. This significant difference was expected as individuals were assigned to the ADHD group based on whether or not they had been previously diagnosed by a physician as having the disorder. It is interesting to note however, that the mean T-scores for the ADHD group did not reach 65 or above on 4 of the 14 measures. Furthermore, there were subjects within the ADHD group who did not present with 65 or higher on any of the scales. Perhaps, these particular individuals have become better able to cope with their symptoms over time, which contributed to the presence of less problematic behaviours. It is also possible that some of these individuals were misdiagnosed with ADHD or "grew out of it". Regardless, had these potentially misdiagnosed individuals not been in the ADHD group here, it is possible that there may have been more significant differences between the groups. There were also subjects in the control group who had multiple scales with T-scores of 65 or more. To this effect, one subject in the control group presented with T-scores greater than 65 on 11 of 14 scales, the fifth most out of all 23 subjects in the study. This same individual was subsequently diagnosed with ADHD shortly after this study ended. As such, all of the data relating to this subject was transferred to the ADHD data pool.

As a group, the ADHD subjects in this study presented with mean T-scores that are comparable to other studies [27-32], although slightly higher on most scales. The higher T-scores in the current study may simply reflect the fact that similar behaviours in a nine-year old would be given a much higher T score, if exhibited by a 13-year old. The behaviour assessment performed here identified that the ADHD group had higher mean T-scores than controls on over seventy-five percent of the Conners' scales, indicating that problematic behaviours persist into adolescence for the majority of these individuals. The Conners' Parent Rating Scale appears to be an extremely reliable tool when comparing ADHD behaviours among individuals of different gender and age.

The red blood cell phospholipid fatty acid composition data reported here, indicates that adolescent ADHD subjects have lower total omega-3 fatty acids, lower DHA levels and higher linoleic acid levels when compared to age-matched control subjects. The absolute values and degree of difference between groups are similar to those reported elsewhere in studies of younger children and adults with and without ADHD[12,30,32,34] However, we did not see several differences reported by Stevens, which included lower AA and adrenic acid levels, and higher docosapentaenoic acid levels in ADHD subjects when compared to controls[29]. Stevens did report lower DHA levels in the ADHD group but this was not statistically significant in their study[29]. In 2004, Chen and colleagues reported significantly lower levels of AA, LA, DHA and total n-3 in red blood cell phospholipids of young children with ADHD when compared to controls[12]. Similarly, when fatty acid composition of red blood cells were compared in an adult population, Young and her colleagues reported significantly lower DHA and significantly higher total n-6 fatty acids among other measures, in the individuals with ADHD[34]. Thus, while there are some differences in the specific fatty acid changes, the data we report here for adolescents indicates that childhood patterns persist through adolescence and into adulthood with few alterations. In several other studies, authors have reported higher AA/EPA ratios in subjects with ADHD. In our study there was a numerical difference but this was not statistically different; a larger sample size may have been able to pick up this difference as the standard deviation for this estimate was quite large. If indeed the levels of AA and EPA are not different between groups, this would suggest that delta-5 desaturase activity may be normal in the ADHD subjects. Rather, the fact that DHA levels were significantly lower in ADHD subjects may suggest a higher rate of oxidation of this fatty acid in these patients. None-the-less, a lower level of circulating DHA may indicate lower levels in the brain as well.

Two previous studies reported dietary patterns in children with ADHD using three day diet records[12,29]. Using a complete seven day diet record, we examined the intake patterns of our adolescent subjects. We demonstrate that ADHD subjects consumed higher levels of at least 10 different nutrients, than their control counterparts. This included 25% more energy, and more grams of carbohydrate, total fat, omega-6 fatty acids and trans fatty acids in addition to others. Stevens reported significantly higher intakes of total fat (g) and polyunsaturated fatty acids (g) in ADHD subjects when compared to control subjects[29]. In 2004, Chen and colleagues reported significantly increased iron (mg) and vitamin C (mg) consumption in subjects with ADHD versus controls[12]. As has been previously proposed, it is possible that over-ingestion of fats could interfere with the conversion of parent essential fatty acids (EFAs) to long-chain(LC)-PUFAs[11]. Despite the many dietary differences between our subject groups, they did not differ in either total omega-3 fatty acid consumption, or consumption of the LC-PUFAs. Thus, it is unlikely that dietary differences in omega-3 consumption can explain the significant differences in red blood cell membrane fatty acids observed in the ADHD subjects herein.

Whatever the reason for lower levels of DHA in ADHD subjects, or abnormal LC-PUFA status, an important question is whether this pattern can be normalized through dietary intervention or supplementation. Early supplementation trials with modest levels (eg. 300–500 mg DHA/day) of LC-PUFAs were largely neutral or showed only modest improvement in biochemical or behavioural parameters in ADHD subjects[27,28,35,36]. However, two recent studies using very high levels of long chain omega-3 PUFAs suggest that the plasma fatty acid abnormalities can be reversed and that there may be significant impact on behavioural parameters [24,25]. Germano and coworkers examined the effects of 0.23 g/kg body weight/day, fish-oil distillate containing a 2:1 ratio of EPA:DHA in 16 ADHD and 31 control children after an 8-week supplementation period[25]. The ADHD group had a significantly higher AA:EPA ratio than the controls at time zero and the supplementation decreased both ratios substantially so that there was no difference between the groups after 8 weeks supplementation. They also reported significant improvements in Inattention and Hyperactivity using the Conner's short-version inventory [25]. Sorgi and colleagues examined the effects of a high dose (initially 16.2 g) EPA/DHA concentrate on nine ADHD children over 8 weeks with dosage adjustment at week 4 depending on AA:EPA ratio [24]. They showed a similar, dramatic decrease in the AA:EPA ratio in the subjects and improvements using the ADHD Symptom Checklist 4, and the short version of the Conners' Parents Rating Scale{Sorgi, 2007 2845/id. These two latest studies suggest that fatty acid supplementation at high levels may be necessary to normalize blood fatty acids, and by assumption brain fatty acids, to achieve improvements in ADHD symptomology. Clearly additional large scale interventions are now warranted.

Regardless of diagnosis in the current study, dietary fat intake was significantly and positively correlated with scores on five of the Conners' scales, positively correlated with total n-6 red blood cell content and negatively correlated with total n-3 red blood cell content. Also, as Chen and colleagues have reported, there was a significantly higher intake of iron in the ADHD group [12]. In the present study, iron intake also displayed a significant and positive correlation to scores on eight of the Conners' scales. It is possible that iron levels in children and adolescents have an effect on behaviours and should therefore be investigated in future studies. Reports have also indicated that children with ADHD may be deficient in magnesium [7,37]. However, in the present study, ADHD subjects reported slightly higher (but not significant) intakes of magnesium when compared to controls. Previous research had also suggested lower intakes of tyrosine, tryptophan and phenylalanine in individuals with ADHD compared to controls[38] however, this was not the case in the current study where again, if anything intakes for these amino acids were higher in ADHD subjects. From these data, a clear role for minerals and amino acids in ADHD behaviours can not be established and will require further investigation.

## Conclusion

This study investigated differences between adolescent populations with and without ADHD with respect to a variety of measures. Despite the small sample size and an imbalance of genders within the groups, several significant differences were reported with respect to overall health, dietary patterns and red blood cell fatty acid compositions. Adolescents with ADHD appear to consume diets more rich in total energy, in addition to specific fats, minerals and other constituents. Although there was no difference in the dietary consumption of n-3 or n-6 fatty acids, adolescents with ADHD did present with significantly lower levels of DHA, total n-3 fatty acids and a lower n-3: n-6 ratio in red blood cell phospholipids. Abnormalites in fatty acid profile were also positively correlated with higher ratings on the Conner's scales. Further research is required to determine the mechanisms by which these fatty acid anomalies occur, and whether indeed supplementation for extended periods with high concentrations of omega-3 fatty acids will positively influence ADHD behaviours in patients of all ages.

## Abbreviations

AA, arachidonic acid; ADHD, attention deficit hyperactivity disorder; ALA, alpha linolenic acid; BIA, bioelectrical impedance analysis; CPRS:L, Conners' Parent Rating Scales Long Version; DHA, docosahexaenoic acid; DGLA, dihomogammalinolenic acid; DRIs, dietary reference intakes; EFA, essential fatty acid; EPA, eicosapentaenoic acid; LA, linoleic acid; LC-PUFA, long chain polyunsaturated fatty acids.

## Competing interests

The author(s) declare that they have no competing interests.

## Authors' contributions

KM conceived of the original idea and aided with the experimental design, writing the final manuscript, data interpretation and provided funding for the study. CC carried out the initial pilot studies on the first few subjects and did much of the background work for the study. AC carried out the all of the subject/parent interviews, collection of biological and behavioural data and all subsequent analysis and assisted with writing of all versions of the manuscript.

All authors have read and approved this manuscript.

## References

[B1] Association AP (2000). Attention deficit hyperactivity disorder. Diagnostic and Statistical Manual of Mental Disorders, Fourth Edition, Text Revision (DSM-IV-TR).

[B2] Harding KL, Judah RD, Gant C (2003). Outcome-based comparison of Ritalin versus food-supplement treated children with AD/HD. Altern Med Rev.

[B3] Scahill L, Schwab-Stone M (2000). Epidemiology of ADHD in school-age children. Child Adolesc Psychiatr Clin N Am.

[B4] Boyle MH, Offord DR, Racine Y, Sanford M, Szatmari P, Fleming JE (1993). Evaluation of the original Ontario Child Health Study scales. Can J Psychiatry.

[B5] Breton JJ, Bergeron L, Valla JP, Berthiaume C, Gaudet N, Lambert J, St Georges M, Houde L, Lepine S (1999). Quebec child mental health survey: prevalence of DSM-III-R mental health disorders. J Child Psychol Psychiatry.

[B6] Rowland AS, Lesesne CA, Abramowitz AJ (2002). The epidemiology of attention-deficit/hyperactivity disorder (ADHD): a public health view. Ment Retard Dev Disabil Res Rev.

[B7] Kidd PM (2000). Attention deficit/hyperactivity disorder (ADHD) in children: rationale for its integrative management. Altern Med Rev.

[B8] Andersen SL, Teicher MH (2000). Sex differences in dopamine receptors and their relevance to ADHD. Neurosci Biobehav Rev.

[B9] Faraone SV (2005). The scientific foundation for understanding attention-deficit/hyperactivity disorder as a valid psychiatric disorder. Eur Child Adolesc Psychiatry.

[B10] Biederman J, Mick E, Faraone SV (2000). Age-dependent decline of symptoms of attention deficit hyperactivity disorder: impact of remission definition and symptom type. Am J Psychiatry.

[B11] Richardson AJ, Puri BK (2000). The potential role of fatty acids in attention-deficit/hyperactivity disorder. Prostaglandins Leukot Essent Fatty Acids.

[B12] Chen JR, Hsu SF, Hsu CD, Hwang LH, Yang SC (2004). Dietary patterns and blood fatty acid composition in children with attention-deficit hyperactivity disorder in Taiwan. J Nutr Biochem.

[B13] Antalis CJ, Stevens LJ, Campbell M, Pazdro R, Ericson K, Burgess JR (2006). Omega-3 fatty acid status in attention-deficit/hyperactivity disorder. Prostaglandins Leukot Essent Fatty Acids.

[B14] Uauy R, Mena P, Rojas C (2000). Essential fatty acids in early life: structural and functional role. Proc Nutr Soc.

[B15] Maida ME, Hurley SD, Daeschner JA, Moore AH, O'Banion MK (2006). Cytosolic prostaglandin E2 synthase (cPGES) expression is decreased in discrete cortical regions in psychiatric disease. Brain Res.

[B16] de Groot RH, Hornstra G, Jolles J (2007). Exploratory study into the relation between plasma phospholipid fatty acid status and cognitive performance. Prostaglandins Leukot Essent Fatty Acids.

[B17] Nakatani Y, Hokonohara Y, Kakuta S, Sudo K, Iwakura Y, Kudo I (2007). Knockout mice lacking cPGES/p23, a constitutively expressed PGE2 synthetic enzyme, are peri-natally lethal. Biochem Biophys Res Commun.

[B18] Innis SM (2007). Fatty acids and early human development. Early Hum Dev.

[B19] Kitaoka S, Furuyashiki T, Nishi A, Shuto T, Koyasu S, Matsuoka T, Miyasaka M, Greengard P, Narumiya S (2007). Prostaglandin E2 acts on EP1 receptor and amplifies both dopamine D1 and D2 receptor signaling in the striatum. J Neurosci.

[B20] Simopoulos AP, Leaf A, Salem N (2000). Workshop statement on the essentiality of and recommended dietary intakes for Omega-6 and Omega-3 fatty acids. Prostaglandins Leukot Essent Fatty Acids.

[B21] Petrie HJ, Stover EA, Horswill CA (2004). Nutritional concerns for the child and adolescent competitor. Nutrition.

[B22] Bjerve KS (1991). Omega 3 fatty acid deficiency in man: implications for the requirement of alpha-linolenic acid and long-chain omega 3 fatty acids. World Rev Nutr Diet.

[B23] McNamara RK, Carlson SE (2006). Role of omega-3 fatty acids in brain development and function: potential implications for the pathogenesis and prevention of psychopathology. Prostaglandins Leukot Essent Fatty Acids.

[B24] Sorgi PJ, Hallowell EM, Hutchins HL, Sears B (2007). Effects of an open-label pilot study with high-dose EPA/DHA concentrates on plasma phospholipids and behavior in children with attention deficit hyperactivity disorder. Nutr J.

[B25] Germano M, Meleleo D, Montorfano G, Adorni L, Negroni M, Berra B, Rizzo AM (2007). Plasma, red blood cells phospholipids and clinical evaluation after long chain omega-3 supplementation in children with attention deficit hyperactivity disorder (ADHD). Nutr Neurosci.

[B26] Mitchell EA, Aman MG, Turbott SH, Manku M (1987). Clinical characteristics and serum essential fatty acid levels in hyperactive children. Clin Pediatr (Phila).

[B27] Arnold LE, Kleykamp D, Votolato NA, Taylor WA, Kontras SB, Tobin K (1989). Gamma-linolenic acid for attention-deficit hyperactivity disorder: placebo-controlled comparison to D-amphetamine. Biol Psychiatry.

[B28] Voigt RG, Llorente AM, Jensen CL, Fraley JK, Berretta MC, Heird WC (2001). A randomized, double-blind, placebo-controlled trial of docosahexaenoic acid supplementation in children with attention-deficit/hyperactivity disorder. J Pediatr.

[B29] Stevens LJ, Zentall SS, Deck JL, Abate ML, Watkins BA, Lipp SR, Burgess JR (1995). Essential fatty acid metabolism in boys with attention-deficit hyperactivity disorder. Am J Clin Nutr.

[B30] Stevens LJ, Zentall SS, Abate ML, Kuczek T, Burgess JR (1996). Omega-3 fatty acids in boys with behavior, learning, and health problems. Physiol Behav.

[B31] Richardson AJ, Puri BK (2002). A randomized double-blind, placebo-controlled study of the effects of supplementation with highly unsaturated fatty acids on ADHD-related symptoms in children with specific learning difficulties. Prog Neuropsychopharmacol Biol Psychiatry.

[B32] Stevens L, Zhang W, Peck L, Kuczek T, Grevstad N, Mahon A, Zentall SS, Arnold LE, Burgess JR (2003). EFA supplementation in children with inattention, hyperactivity, and other disruptive behaviors. Lipids.

[B33] Conners CK (1997). Conners' Rating Scales-Revised: Technical Manual.

[B34] Young GS, Maharaj NJ, Conquer JA (2004). Blood phospholipid fatty acid analysis of adults with and without attention deficit/hyperactivity disorder. Lipids.

[B35] Aman MG, Mitchell EA, Turbott SH (1987). The effects of essential fatty acid supplementation by Efamol in hyperactive children. J Abnorm Child Psychol.

[B36] Hirayama S, Hamazaki T, Terasawa K (2004). Effect of docosahexaenoic acid-containing food administration on symptoms of attention-deficit/hyperactivity disorder - a placebo-controlled double-blind study. Eur J Clin Nutr.

[B37] Kozielec T, Starobrat-Hermelin B (1997). Assessment of magnesium levels in children with attention deficit hyperactivity disorder (ADHD). Magnes Res.

[B38] Bornstein RA, Baker GB, Carroll A, King G, Wong JT, Douglass AB (1990). Plasma amino acids in attention deficit disorder. Psychiatry Res.

